# Automated Assessment of the Quality of Phonocardographic Recordings through Signal-to-Noise Ratio for Home Monitoring Applications

**DOI:** 10.3390/s21217246

**Published:** 2021-10-30

**Authors:** Noemi Giordano, Samanta Rosati, Marco Knaflitz

**Affiliations:** Department of Electronics and Telecommunications and PoliToBIOMedLab, Politecnico di Torino, Corso Duca degli Abruzzi 24, 10129 Torino, Italy; samanta.rosati@polito.it (S.R.); marco.knaflitz@polito.it (M.K.)

**Keywords:** phonocardiography, signal-to-noise ratio, heart failure, heart valve timing, automated quality assessment, telemonitoring

## Abstract

The signal quality limits the applicability of phonocardiography at the patients’ domicile. This work proposes the signal-to-noise ratio of the recorded signal as its main quality metrics. Moreover, we define the minimum acceptable values of the signal-to-noise ratio that warrantee an accuracy of the derived parameters acceptable in clinics. We considered 25 original heart sounds recordings, which we corrupted by adding noise to decrease their signal-to-noise ratio. We found that a signal-to-noise ratio equal to or higher than 14 dB warrants an uncertainty of the estimate of the valve closure latencies below 1 ms. This accuracy is higher than that required by most clinical applications. We validated the proposed method against a public database, obtaining results comparable to those obtained on our sample population. In conclusion, we defined (a) the signal-to-noise ratio of the phonocardiographic signal as the preferred metric to evaluate its quality and (b) the minimum values of the signal-to-noise ratio required to obtain an uncertainty of the latency of heart sound components compatible with clinical applications. We believe these results are crucial for the development of home monitoring systems aimed at preventing acute episodes of heart failure and that can be safely operated by naïve users.

## 1. Introduction

Heart sounds are acoustic waves mainly generated by the closing of heart valves [1]. Their noninvasive recording by means of a microphone gives origin to a biomedical signal known as phonocardiogram (PCG). Even though traditional auscultation has suffered a decline over the years due to its lack of objectivity, its digital counterpart is growing in importance [2]. PCG analysis was proved as a valuable tool for the follow-up of patients affected by cardiovascular diseases (CVDs), the first world cause of death according to the World Health Organization [3].

In a previous study [4], we presented an automated algorithm for the segmentation of heart sounds within a PCG recording and for the user independent measurement of the timing of heart sound components: mitral and tricuspid components in first heart sound (S_1_), and aortic and pulmonary components in second heart sound (S_2_). The knowledge of the latency of heart sound components is helpful in studying the electromechanical coupling of the heart muscle fibers of a subject and consequently a variety of pathologies, such as heart failure [4,5,6,7,8].

The recent n-SARS-CoV-2 pandemic had a tremendous impact to homecare, due to the need of reducing as much as possible patient access to hospitals. This tragic situation forced many countries to speed up the development of homecare services, particularly for patients affected by chronic diseases or recovering after surgery or severe acute events. At this time, the need for devices and techniques developed for naïve users, able to collect reliable information at the patient domicile and, when necessary, to transfer data to medical centers or physicians providing homecare services, is widely recognized.

In developed countries, 20% of healthy people between 40 and 50 has a high probability of developing a heart failure condition in the elderly. The prevalence of heart failure in Europe is approximately 2% [9], while incidence is approximately 0.30% in Sweden [10].

In the field of signal processing, a typical problem is to evaluate which constraints an algorithm is subjected to and in which conditions it provides reliable results. This is crucial in the biomedical area because improper use of an algorithm may lead to incorrect results and hence to a wrong evaluation of the patient’s conditions, which could negatively affect the treatment planning and outcomes.

This issue becomes particularly critical when users are unskilled (naïve users), as in domiciliary applications [11,12]. In a home monitoring context, the availability of a robust, automated, easy-to-use tool for the assessment of the quality of the estimation of the closure timing of the heart valves would tremendously increase the possibility of correctly evaluating the status of the patient’s heart conditions. This would enable the early treatment of pathologies related to valvular abnormalities and acute episodes of heart failure [13].

Defining the minimum signal-to-noise value (SNR) necessary to obtain accurate results appears of key importance for delimiting the field of use and the range of applicability of an algorithm [11,14].

To date, a critical aspect that limits the applicability of clinical phonocardiography is the technical difficulty of acquiring high-quality PCG signals. This is due to two main reasons:

- The quality of the recordings strongly depends on the positioning of the digital stethoscope [15,16,17,18].
- An impressive amount of noise and artifacts affects PCG recordings [19].

**Figure 1:**
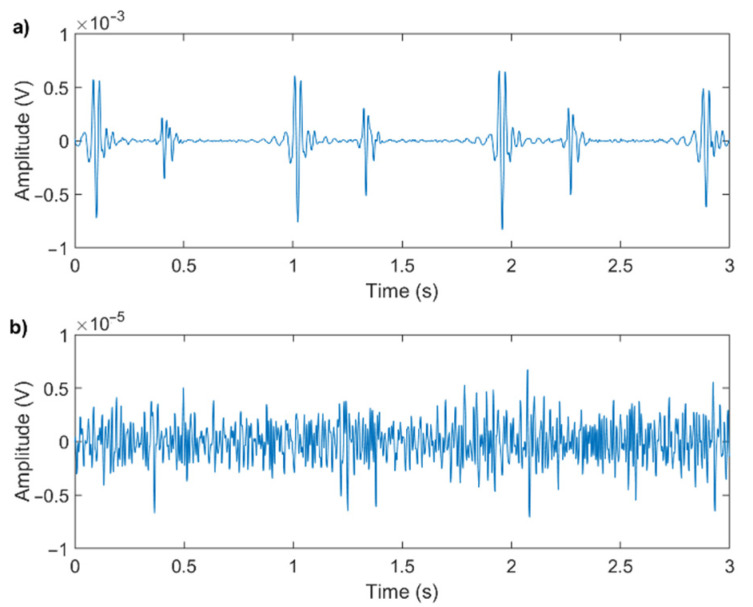
Comparison between (**a**) a good-quality signal (SNR = 31 dB) and (**b**) a poor-quality signal (SNR = 4 dB). The extraction of valve timing and even the SNR computation is evidently questionable in case (**b**).

To provide reliable results, algorithms for heart sound segmentation and classification require a good-quality PCG signal [19]. The application of heart-sound processing algorithms to poor-quality signals may lead to diagnostically misleading results [12,14]. Figure 1 shows an example of a good-quality and a poor-quality signal: the two signals were recorded in similar conditions, and both have been digitally filtered for noise reduction.

Even though the recording conditions and processing methods are equal for the two signals, their quality strongly depends on the physical characteristics of the subject and on the positioning of the microphone. It is evident that trying to extract valve timing information from the poor-quality signal would be impossible.

The aim of this work is to evaluate the sensitivity to noise of an algorithm we previously developed [4]. Specifically, in this work, we present the minimum SNR values of the signals to be processed that grant an accuracy of the valve timing estimate adequate for different clinical needs. A ready application of this study is the possibility of developing an automated tool for performing a real-time quality assessment of PCG signals.

## 2. Materials and Methods

This section presents the rationale of this study and shows the metrics we used to assess the signal quality and to evaluate the reliability of the results of the algorithm. In the end, we describe the sample and the validation populations used to test and validate the presented methodology.

### 2.1. Rationale

In this work, we used real PCG recordings with original SNR values ranging from 7 dB to 25 dB (computed with the methodology presented in Section 2.2) and we progressively reduced the original SNR by adding a noise process with increasing power. Recordings were performed on healthy subjects in a laboratory setting.

The following equation shows the model of the signal corrupted by the original noise and by the additive noise, artificially introduced to decrease the SNR:

(1) $$x\left(t\right)=s\left(t\right)+n\left(t\right)+n_{i}\left(t\right),$$

where s(t) is the original acoustic signal as emitted by the heart, before any noise is superimposed; n(t) is the additive noise originally superimposed to the signal in the recording phase (the sum of s(t) and n(t) is the actual recording); $n_{i}\left(t\right)$ is the noise artificially added to the original signal to decrease its SNR; x(t) is the resulting signal, that was used to compute the latencies of the heart sound components.

The original signal s(t) and the noise originally superimposed n(t) were assumed as uncorrelated. The noise process $n_{i}\left(t\right)$ was generated as a white stochastic process uncorrelated to s(t) and n(t). We modeled the noise process $n_{i}\left(t\right)$ as a Gaussian white stochastic process because we found the noise originally superimposed n(t) to present a normal distribution with a zero mean in the large majority of heartbeats. We applied the Chi-square goodness of fit test to the segment between 70% and 85% of each cardiac cycle when no heart sound is expected to occur [1], against the normal distribution, which we hypothesized as a suitable noise model (α = 0.05). Over more than 16,000 heartbeats, the null hypothesis, stating that the samples come from a normal distribution, could not be rejected in 84% of the noise segments. Therefore, a Gaussian white stochastic process was found as a suitable noise model.

From each original recording, we obtained a set of signals in which SNR progressively decreased by 0.1 dB steps. Specifically, for each signal $x_{i}\left(t\right)$, additive noise $n_{i}\left(t\right)$ was generated with a standard deviation equal to:

(2) $$\sigma_{i}=\sigma_{0}\;\cdot \;\sqrt{10^{\;\frac{0.1\cdot i}{10}}-1},$$

where $\sigma_{0}$ is the average standard deviation of noise in the original recording, computed within 70% and 85% of the cardiac cycle when no heart sound is expected to occur [1]. To segment the PCG signals into cardiac cycles, R-peaks of a simultaneous electrocardiogram were used as reference.

**Figure 2:**
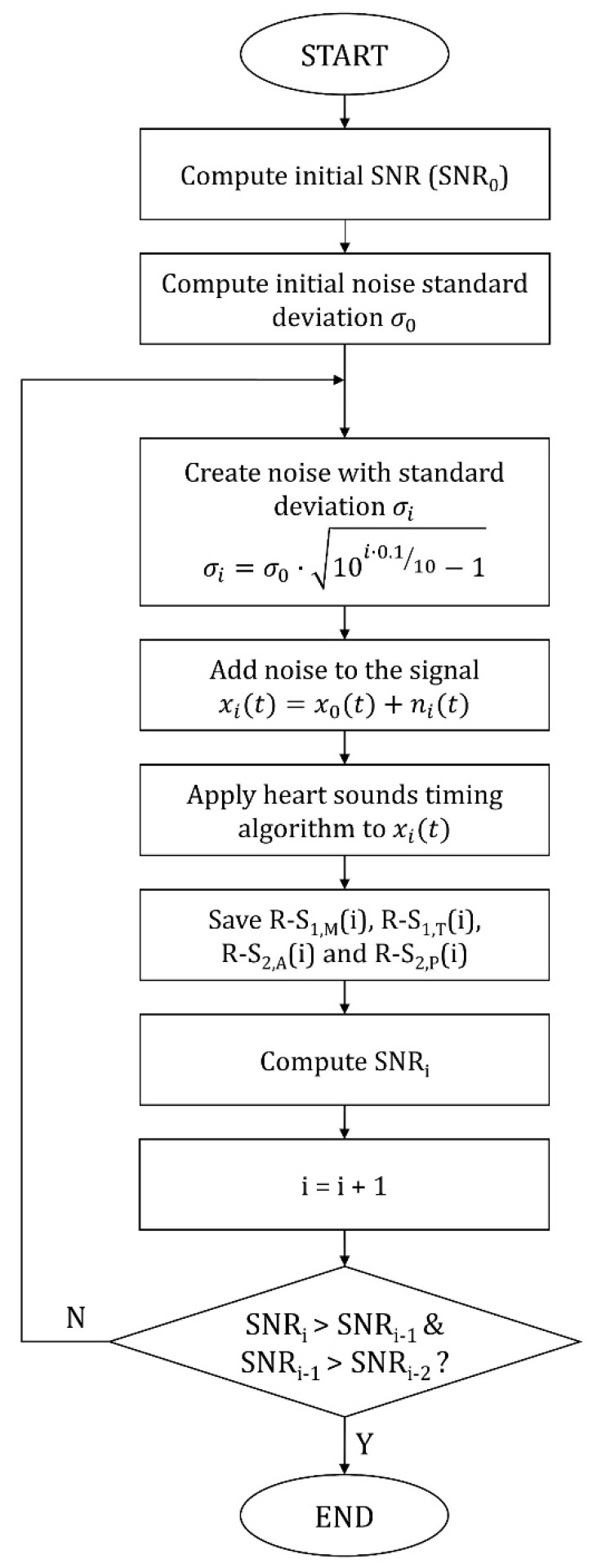
Flowchart of the algorithm described in this work. R-S_1,M_, R-S_1,T_, R-S_2,A_ and R-S_2,P_ are the latencies of the mitral, tricuspid, aortic and pulmonary valves with respect to the corresponding R-peak.

The SNR is expected to decrease by approximately 0.1 dB at each iteration. The computation of SNR relies on the correct identification of heart sounds within the signal: when the signal quality is very poor, the resulting SNR value may be inaccurate. Therefore, when the computed value of SNR stops decreasing in two consecutive iterations, the stop condition is met. Figure 2 represents the flowchart of the process.

### 2.2. PCG Quality Assessment

Few methods for PCG quality assessment were previously proposed in the literature, as expanded in Section 4. In most cases, it was suggested to assess the quality of a PCG signal based on its physiological characteristics, by extracting a set of features from the signal itself, which are then used to feed a machine learning algorithm or to build some criteria.

In this study, we propose to evaluate the signal quality by means of elementary numerical methods applicable to the single heartbeat. Our purpose is to have a quality evaluation that does not depend on signal features that may vary in case of pathological conditions. Moreover, we want to evaluate the signal quality directly on the raw recordings, prior to any filtering process. Our aim was to develop a straightforward methodology to assess the signal quality in real time, to provide inexperienced users with direct feedback, easy to understand. This requires a quality assessment algorithm easily implementable onboard of the recording system: it follows that simple numerical computations are preferable.

Moreover, a robust evaluation of the quality of the recordings is of key importance for use in telehealth applications, where no human feedback is available.

Consequently, we performed PCG quality assessment only based on the SNR of PCG, which takes into account the level of noise of the recorded signal and is independent of its characteristics and on the presence of any pathological state.

In particular, we computed the SNR value separately for the first and second heart sounds, thus obtaining two SNR values for each heartbeat.

We assume PCG signals as quasi-periodic and the additive noise as a stochastic process. Therefore, we defined SNR as in Equation (3):

(3) $$SNR=20\;\log_{10}\frac{A_{S}}{4\;\sigma_{N}},$$

where

- $A_{S}$ is a measure of the signal amplitude, defined as the peak-to-peak amplitude of the heart sound of interest;
- $4\;\sigma_{N}$ is a measure of the noise amplitude, corresponding to the amplitude of the 95% band of noise. This is assumed as having a normal probability density function of its amplitude. The latter is computed within 70% and 85% of the heart cycle when no heart sound is expected to occur [4].

### 2.3. Criteria for the Evaluation of the Results

To assess the robustness of the algorithm towards the noise, we quantified the variations of the latencies of the two components of both S1 and S2 as a function of a progressive decrease in the signal SNR.

Specifically, we considered:

- R-S_1,M_: the latency between the mitral component of the first heart sound and the corresponding R-peak;
- R-S_1,T_: the latency between the tricuspid component of the first heart sound and the corresponding R-peak;
- R-S_2,A_: the latency between the aortic component of the second heart sound and the corresponding R-peak;
- R-S_2,P_: the latency between the pulmonary component of the second heart sound and the corresponding R-peak.

**Figure 3:**
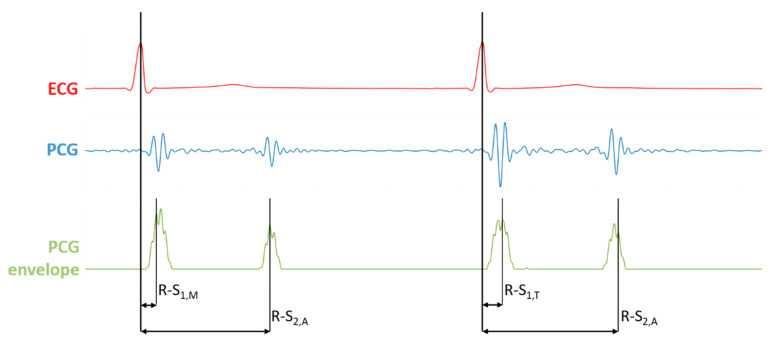
Visual representation of the latencies R-S_1,M_, R-S_1,T_, R-S_2,A_ and R-S_2,P_ as extracted from ECG and PCG simultaneous signals.

The technique for the estimation of the latencies is presented in a previous work [5]. Figure 3 shows a visual representation of the latencies.

**Figure 4:**
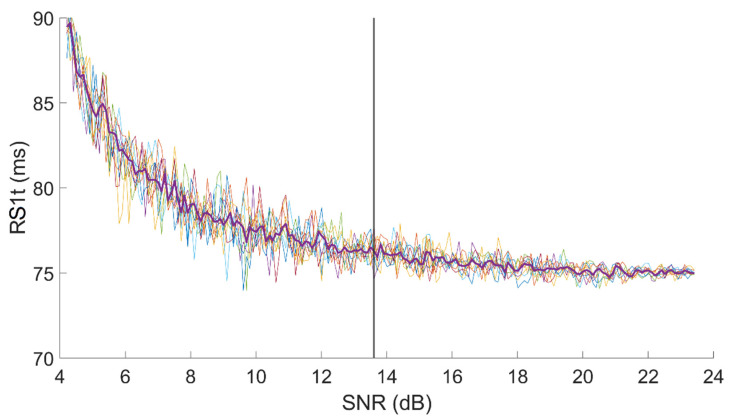
Example of R-S_1,T_ variation in function of the SNR of the signal. Each colored line represents a different test on the same signal. The thicker violet line represents the median, which is taken as representative for the signal. The vertical line represents the minimum SNR value considered as acceptable.

Figure 4 shows an example of the dependency of R-S_1,T_ on SNR in a signal with an original SNR, for first heart sound, equal to 24 dB.

The test was repeated 10 times on every signal. For each test, we computed the value of each latency for each SNR value. Thus, we obtained 10 curves similar to those presented in Figure 4. In the end, we resampled all 10 curves at fixed SNR points, keeping the resolution equal to 0.1 dB, and we computed the median curve, which we consider as representative for the single subject.

Finally, we computed the minimum acceptable SNR value as the value that corresponds to the first measurement of the latency, in decreasing SNR order, which significantly differs from the initial (true) value. We considered as “significantly different” a value for which two conditions were met:

1. Presence of a trend. It was identified by applying Wald–Wolfowitz runs test (α = 0.05) [20];
2. Difference from the reference higher than 1 ms, i.e., higher than the resolution of the acquisition system.

In Figure 4, a vertical line shows the minimum SNR value we may accept on the sample R-S_1,T_ set of curves assuring a 1 ms resolution.

The criteria adopted grant the same resolution that the acquisition system offers (1 ms). Nevertheless, in the clinical context, in most cases, a higher measurement uncertainty could be accepted.

Therefore, we computed the minimum acceptable SNR again, with the same methodology described above, for different time-resolution values. The scope is to allow for considering the minimum SNR value that may be acceptable, depending on the clinical application.

We repeated the computation by considering as measurement uncertainty subsequently higher percentages of the mean value of the latency over the sample population (2.5%, 5%, 7.5%, 10%, 12.5%, 15%, 17.5%, 20%).

We believe that a measurement with an uncertainty higher than 20% of its value is too rough for any kind of clinical application. In fact, when considering the first heart sound, which commonly shows a split as long as 20–30 ms in healthy subjects [4,21], a measurement uncertainty of more than 20% over values of approximately 40–50 ms [4,22] could negatively affect the capability of the algorithm of distinguishing between the mitral and the tricuspid components in several subjects.

### 2.4. Sample Population

The sample population for this study consisted of 25 healthy volunteers. The study was retrospectively conducted based on the signals recorded for the previous study we published in [4]. The experimental protocol conformed to the Helsinki declaration and subjects signed an informed consent form. For each subject, we recorded 10 min of PCG and ECG signals.

We performed recordings using a commercial acquisition system for biomedical signals (ReMotus^®^, It-MeD, Torino, Italy) and a custom-made microphone probe based on a condenser electret microphone.

A more detailed description of the technical features of the acquisition system can be found in [4]. A recording from the sample population is provided as example in the Supplementary Material, along with 5 versions of the same recording where noise was added to decrease its SNR value down to respectively 25 dB, 20 dB, 15 dB, 10 dB, 5 dB. 

### 2.5. Validation against a Publicly Available Database

To validate the proposed methodology and the resulting minimum acceptable SNR values, we relied on a public database. For this purpose, we used the PhysioNet CinC Challenge 2016 database, which comprises recordings from eight publicly available heart sound databases [23]. Among the available recordings, only those from the Massachusetts Institute of Technology heart sound database (training set “A”) had a simultaneous recording of an ECG associated, which is a fundamental requirement for the scopes of our method. Therefore, we selected the recordings from the MIT database and extracted the ones classified as “Normal”, i.e., from patients with no history of cardiopathy, to match the characteristics of our sample population. Of this database, 117 recordings satisfied the requirements.

The MIT database is particularly suitable for the validation of the proposed methodology for the PCG quality assessment because the recordings were performed in an uncontrolled environment and, partially, in a domicile context. Therefore, the phonocardiograms are affected by a large variety of noise contributions. On the other hand, the ECG signals were not always good-quality ones: sometimes their SNR or morphology were not sufficient to accurately identify the R-wave peak to be used as a reference for the estimation of the latencies. For this reason, we discarded the recordings not satisfying the following criteria:

1. The amplitude of the R-peak must be at least three times wider than the amplitude of the noise 95% band (14 signals were discarded).
2. The first high-amplitude wave of the QRS complex (R-wave) must have the same polarity as the T-wave (15 signals were discarded).

In this way, we used 88 out of 117 recordings. In the following, “sample population” will refer to our recordings, which we used to build and test the proposed method for PCG quality assessment, whereas “validation population” will refer to the population used for the validation of the method, which we extracted from the MIT database.

The validation was carried out by comparing the minimum acceptable SNR value obtained for each of the recordings of the validation population to the minimum acceptable SNR values computed for the sample population. To better match our database, only recordings with an original SNR before corruption higher than 14 dB were used (42 signals). We used the percentage of recordings with a minimum acceptable SNR value lower or equal to the one computed over the sample population as metrics to verify the robustness of the methodology.

## 3. Results

In this section, first, we present the minimum SNR values considered as acceptable respectively for the latency of the mitral, tricuspid, aortic, and pulmonary components. These values were computed to grant a measurement uncertainty of the latencies equal to the resolution of the acquisition system (1 ms).

Then, we show how the minimum SNR values change when an increasing uncertainty may be acceptable, as often happens in clinical conditions.

### 3.1. Minimum Acceptable SNR Values with 1-ms Uncertainty

Table 1 presents, for each subject, the initial SNR value for each heart sound, which is an index of signal quality, and the computed minimum value of SNR considered adequate for the estimation of each latency parameter. The initial SNR values (SNR_1_, SNR_2_) are computed on the raw PCG signal, before applying any digital filtering.

It should also be highlighted that, in some cases, all the computed values differed less than 1 ms from the reference value, for any SNR value. Therefore, in those cases no threshold could be computed and, in Table 1, they are reported as “N/A”.

The minimum acceptable SNR values over the population are computed as the maximum acceptable value of SNR found over the entire group of subjects for each parameter (worst case) and are shown in the last row of Table 1.

Observing the results, we can conclude that raw recorded signals with an original SNR value higher or equal than 14 dB for both heart sounds may be considered as adequate for estimating the latency of heart sound components with an uncertainty of 1 ms.

It must be emphasized that signals which are considered below threshold in this step are not necessarily inadequate for clinical applications.

**Figure 5:**
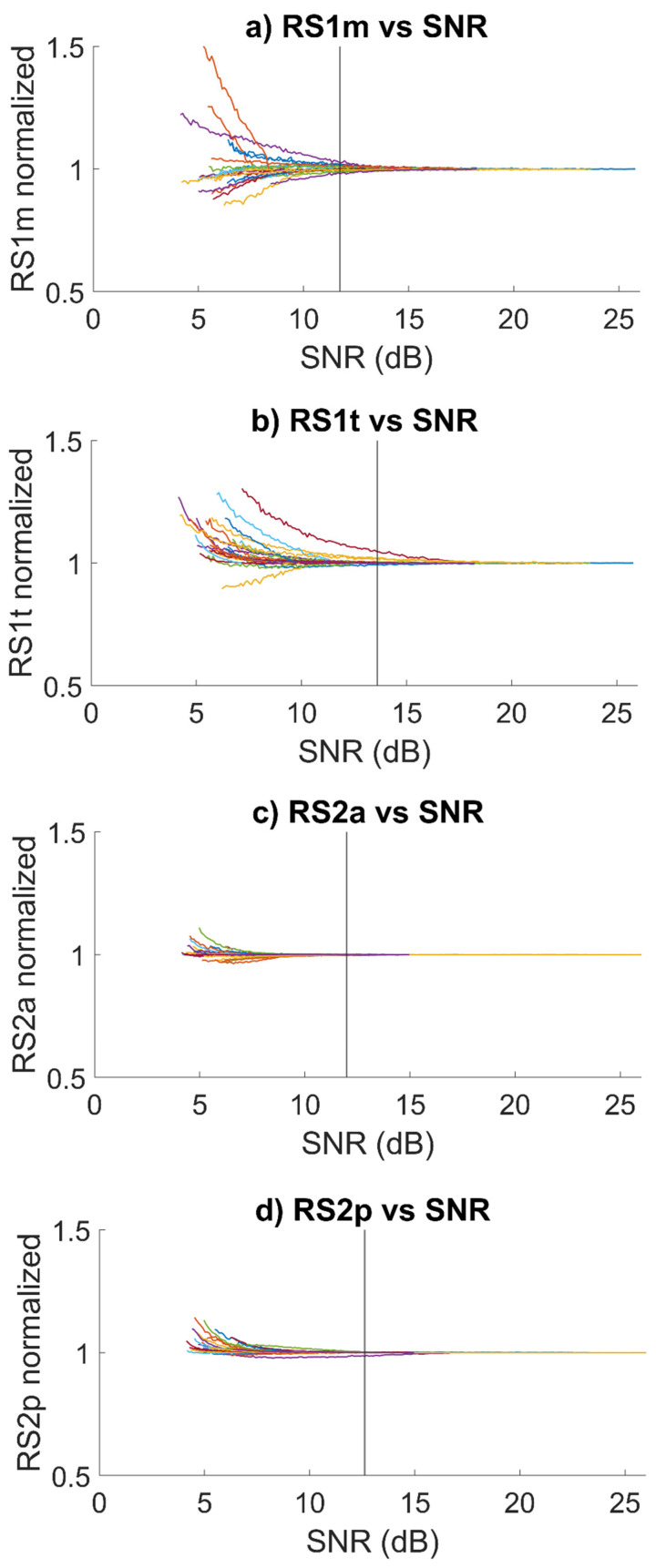
Plots of respectively R-S_1,M_, R-S_1,T_, R-S_2,A_, and R-S_2,P_ in function of the SNR of the signal for all 25 subjects. All values were normalized with respect to the value corresponding to the highest SNR for the purposes of comparison. The black vertical lines represent the computed minimum acceptable SNR values. Each colored line represents a subject from the sample population.

Figure 5 presents the plots of respectively R-S_1,M_, R-S_1,T_, R-S_2,A_ and R-S_2,P_ in the function of the SNR of the signal for all 25 subjects. All values were normalized with respect to the value corresponding to the highest SNR for the purposes of comparison. Each colored line represents a subject, whereas the vertical line is the minimum acceptable SNR value for the population.

Figure 5 shows that, even at SNR values lower than the computed threshold, the variation of the latency with respect to the reference value is not particularly relevant, especially when considering the second heart sound.

### 3.2. Minimum Acceptable SNR Values as Function of Uncertainty

In a clinical context, the uncertainty considered acceptable is usually expressed as a percentage of the value of the measurement.

In this study, we examined the effect of uncertainties ranging from 2.5% to 20% of the latency estimate, which may be considered reasonable in most clinical applications. For each heart valve, the minimum and maximum latencies $RS_{x,y\;THS}$ (with x=1, 2 and y=M, T, A, P) that give the percentage uncertainty $\varepsilon_{\%}$ were computed according to Equation (4):

(4) $$RS_{x,y\;THS}=RS_{x,y\;REF}\;\pm \;\frac{\varepsilon_{\%}}{100}\;*\;RS_{x,y\;REF},$$

where $RS_{x,y\;REF}$ is the reference latency, i.e., the median of the latency computed over the sample population. Table 2 reports the median values (along with 25th percentile and 75th percentile values) for the four latencies over the sample population, used as references to compute the percentage uncertainties.

**Figure 6:**
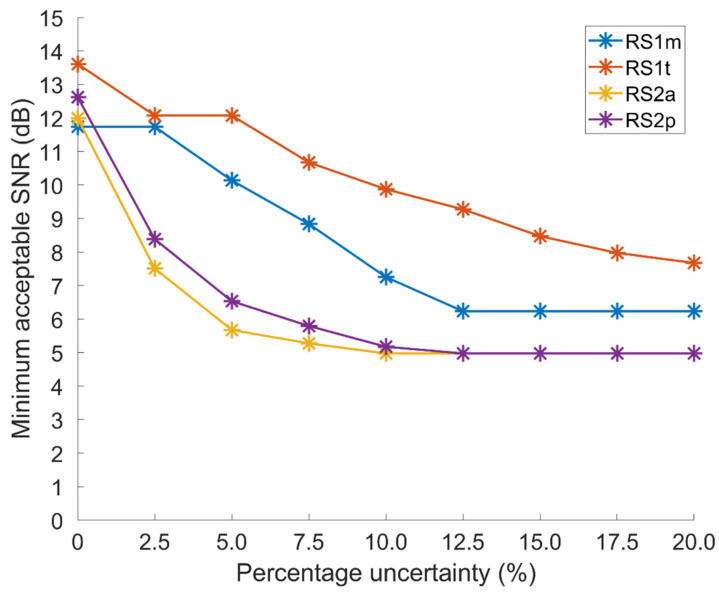
Minimum acceptable SNR for the estimation of respectively R-S_1,M_, R-S_1,T_, R-S_2,A_, and R-S_2,P_ as a function of the acceptable percentage uncertainty over the measurement of the hearth valves closure latencies.

The minimum acceptable SNR value is the SNR corresponding to the first latency outside the range in decreasing SNR order. Figure 6 presents the minimum SNR as a function of the measurement uncertainty considered as acceptable. The reported values are the maximum values found over the sample population (worst case).

The estimates of the latencies of S_1_ components are more sensitive to noise than those of S_2_ components. Moreover, the measurement of the closing time of the valves of the left side of the heart is more robust towards SNR than their counterpart in the right heart, especially in the case of atrioventricular valves. This makes sense since left heart pressure gradients are higher than right heart ones.

For the second heart sound components, it was not possible to define a minimum acceptable SNR value for high uncertainties (higher than 10% for aortic, higher than 12.5% for pulmonary valves). In fact, in the tested conditions, the estimated latencies never differ from the initial value by more than 10% and 12.5%, respectively. Therefore, the minimum SNR reached was chosen as the minimum acceptable SNR value. For the second heart sound components, it is possible to obtain a 5% uncertainty with signals showing an SNR as low as 6 dB, which may be obtained on almost every subject with a good acquisition system and an experienced user.

### 3.3. Results of the Validation against a Public Database

**Figure 7:**
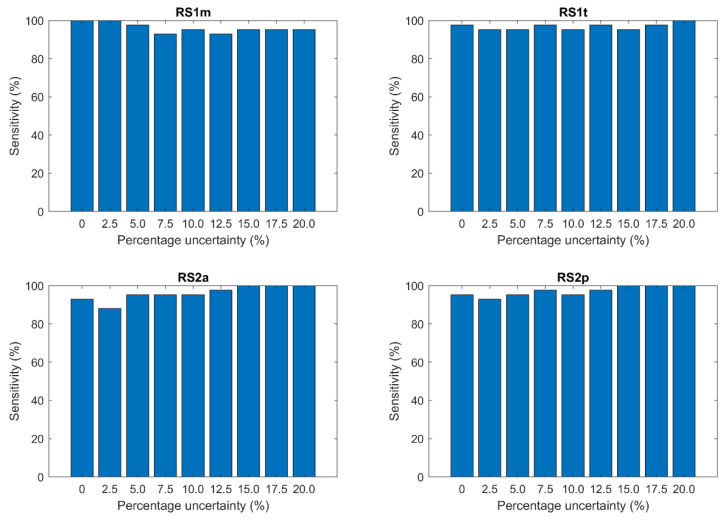
Results of the validation. Each bar represents the sensitivity of the method for a specific acceptable percentage uncertainty, i.e., the number of recordings of the validation population that satisfies the corresponding percentage uncertainty when their SNR is decreased down to the minimum acceptable value defined on the sample population.

As mentioned in Section 2.5, we evaluated the robustness of the computed minimum SNR values as a function of the acceptable percentage uncertainty against recordings from a publicly available dataset. Figure 7 presents the results.

The height of each bar in Figure 7 represents the percentage of recordings from the validation population that satisfies the corresponding percentage uncertainty when their SNR is decreased down to the minimum acceptable value defined above (Figure 6) on our database.

It should be highlighted that, differently from Section 3.2, the “0” column corresponds to an absolute uncertainty smaller than the 95% confidence interval (CI95) of the estimate of the latency, instead of the 1 ms resolution of the acquisition system. This is due to the fact that the duration of the recordings belonging to the validation population (12 to 36 s) is one order of magnitude shorter than that of the recordings belonging to the sample population (10 min). Since the estimate of the latency is computed as the average of the latency values over the recorded heartbeats, the availability of a significantly lower number of heartbeats negatively affects the robustness of the estimate.

## 4. Discussion

Monitoring of patients suffering from a form of cardiomyopathy that makes them exposed to episodes of acute heart failure is of paramount importance to avoid the acute episode and the hospitalization of patients. It is well known that each acute episode significantly compromises the life expectance of the patient [24,25]. Approximately 10–20% of patients that develop an acute episode will die within 30 days [25], and, considering a period of 5 years from the first acute episode, only 50% of patients will survive [24]. In developed countries, the prevalence of chronic heart failure is approximately 1% in the population aged from 54 to 65 years, it increases up to 3% between 65 and 74 years, shows a further increase to approximately 7% from 75 to 84 years, and reaches a percentage as high as 10% for people over 84 years of age [25]. At this time, people aged from 40 to 50 years have a probability of developing a form of chronic heart failure up to 21% all during their life, and this probability increases up to 28% for forty-year-old people affected by a form of hypertension [25].

Approximately 1–2% of the costs of health expenses are due to cardiac heart failure [26], and approximately 60% of these costs is due to hospitalization [26,27]. On the average, an acute episode requires one week hospitalization of the subject, that, in turns, costs over USD 10,000 in the U.S. [27,28], as well as in Europe.

These data suggest that inexpensive methods and devices for preventing acute episodes of cardiac heart failure would warrantee a considerable increase in active life expectancy to predisposed people while allowing sanitary systems to save a large amount of money. A typical win–win approach.

For patient monitoring to be effective, tolerable, and low cost, it should be based as much as possible on the monitoring of compensated patients, which are known to be exposed to episodes of cardiac acute heart failure. Monitoring should be performed at their domicile by means of inexpensive, reliable, and easy-to-use devices. A naïve user, typically the patient or a caregiver, should be able to operate these monitoring devices, which should be able to identify elevated risk conditions and to suggest patients to contact their cardiologist. A set of significant data should be transferred by the system at the patient’s domicile to the physician in charge, to allow her/him to take proper countermeasures to prevent the acute episode.

Platforms allowing for the follow-up of patients prone to cardiac failure at their domicile are already in use, they are generally recognized as useful [29,30], but their effectiveness still needs to be improved.

From a physiological point of view, an episode of heart failure is most frequently generated by a decreased pumping capacity of the cardiac muscle, often due to a decrement in the effectiveness of the electro-mechanical coupling in ventricular muscle fibers. In turn, a decrement of the contractile force of ventricles causes a delay in the closure times of the heart valves, measured, generally, taking the R-wave (ventricular depolarization) as a reference. It has been demonstrated that diminished effectiveness of heart pumping is reflected in an increment in the closure latencies of the heart valves. Moreover, the possibility of measuring the specific latency between the R-wave and the closure of each heart valve also allows for discriminating among different forms of heart failure (i.e., left heart failure, right heart failure, or a failure affecting both heart ventricles).

To the best of our knowledge, at this time, there is a single report in the literature that describes how to extract the latencies of the closure of the four heart valves with reference to the depolarization of ventricles [4] without resorting to cardiac catheterization. Though the cited methodology has been proven as effective in a normal population, no data are currently available to predict the accuracy of the estimated latencies as a function of the signal quality. In fact, the quality of PCG signals is very variable also in normal subjects and may be further compromised in subjects that are developing an acute form of heart failure. Hence, to maximize the reliability of follow-up procedures at the patient domicile by means of devices based on PCG signals, it is of paramount importance to define a metric that allows to automatically detect when the quality of the recorded signal is not sufficient for its clinical interpretation. It must also be considered that the different clinical conditions of different patients may be compatible with different accuracies of the latencies of the closure of valves with respect to the ventricular depolarization. Hence, the physician should be allowed to choose an accuracy of the estimates that fits the conditions of each specific patient.

In the literature, methods for PCG quality assessment are not abundant, although in the latest years this issue gained an increasing interest within the scientific community.

A first family of proposed approaches, published approximately ten years ago, based the quality assessment on the periodicity of the PCG signal. Li et al. [31] proposed an automated algorithm to select a subset of subsequent heartbeats based on their degree of periodicity. Kumar et al. [32] based their quality assessment on the periodicity of heartbeats, by selecting a heartbeat characterized by a low noise level within the recording and comparing the remaining ones with it by spectral matching. A good-quality signal was characterized by a high level of matching.

Several Authors reported different methods involving a wider set of physiological features extracted from the signal in the time, frequency, or time-frequency domains. Some Authors combined the selected features into the evaluation criteria. Naseri et al. [11] proposed a quality assessment method that relies on features such as the logarithmic energy of the signal, the level of noise outside the typical bandwidth of heart sounds and murmurs (>700 Hz), and the duration of the segments corresponding to the heart sounds. Grzegorczyk et al. [33] used three criteria based on the root mean square of successive differences of the signal and the number of detected peaks over different moving windows. More recently, Mubarak et al. [19] relied on voting among three criteria, which were the root mean square value of successive differences between heart sounds, the ratio of zero crossings in the signal, and the ratio of segments having a “normal” number of peaks.

In the latest years, different Authors proposed several methods based on machine learning which considered a large number of features. These methods typically define a recording in a binary way, as either of sufficient quality or not. This kind of output is very easy to interpret and is also well accepted by clinicians. Its limitation is represented by the fact that it does not make available numerical quality metrics. Zabihi et al. [34] proposed a quality assessment method based on an ensemble of 20 Neural Networks, fed with 40 features from the time, frequency, and time-frequency domains. It should be highlighted that in later work the same authors used the SNR of the PCG signal to divide their dataset into good vs. poor quality signals as a preprocessing step [35]. Springer et al. [12,36] proposed nine signal quality indices, mostly based on the autocorrelation of the PCG envelope, which they used to feed a support vector machine [36] and a logistic regression classifier [12]. Very recently, Shi et al. [14] developed a method for assessing the quality of heart sounds within radar-recorded signals. They proposed ensemble classification on a wide range of extracted features, which they considered as a complement to Springer’s quality indices [12]. They used the SNR of the signals, labeled as high-quality or low-quality by a medical staff through visual inspection, as a reference for validation. An obvious limitation of this approach is the need for expert operators to classify each single signal recording. Also in 2020, Chakraborty et al. [37] published a work in which they used a convolutional neural network fed with the spectrogram of the PCG signal to evaluate the signal quality. In the same year, a paper by Grooby et al. [38] proposed the extraction of as many as 187 features from neonatal PCG signals and classified their quality using an ensemble classifier combining a support vector machine, a decision tree, K-nearest neighbors, and a Gaussian Naïve Bayes classifier. A major limitation of this approach is the difficulty in relating most of the 187 features to cardiac physiology or to visually inspect signal features easily, thus binding the user to completely trust the methodology.

Most of the reviewed methodologies presented in the last five years imply the usage of a wide range of features extracted from the signal, which are correlated to the morphology of the signal, sometimes not so obvious. This makes it difficult for physicians to understand why a specific signal is not considered of sufficiently quality, which is information that could be relevant from a clinical point of view. Moreover, a large number of signal features, even if physiological interpretation is not obvious, may be affected by the presence of a pathological condition.

In this paper, we present a metric that is very easy to interpret and that may be, if necessary, visually inspected in a very simple way by clinicians. A second very important advantage of the usage of a numerical metric such as the SNR is that its meaning and interpretation are independent of the health status of the subject.

Section 3 shows that SNR is a very robust metrics to assess the quality of a PCG recording. As the monotonic trend in the curves of Figure 5 shows, the amount of error in the estimation of the latency of heart sound components appears positively correlated to the decrease in the SNR of the signal, as expected. We believe that relying on an SNR-based quality assessment of the PCG signal quality grants direct feedback regarding the reliability of results obtained by home monitoring systems.

As highlighted in the Results session, we found that an SNR as low as 14 dB is sufficient for obtaining an estimate of the closure latencies of hearth valves with an uncertainty lower than 1 ms, which is definitely higher than that generally required in clinics. By means of the algorithm, we previously published [4], phonocardiography recordings of such quality could be obtained with an average electronic stethoscope, also at the patient’s domicile, as soon as other technical problems (e.g., the positioning of microphones over the chest) are solved.

We already stated that, to our best knowledge, at this time there is no other possibility for obtaining a day-to-day follow-up of the timing of the closure of heart valves with non-invasive techniques, in residential environments, and without requiring the intervention of an expert user with a clinical background. This is an important point of strength of this technique.

The second strength of our approach is represented by the possibility of adapting the minimum acceptable SNR value to the clinical needs of the specific patient. In fact, since an estimation accuracy as high as 1 ms is generally unnecessary in most clinical situations, Figure 6 shows that it is possible to adapt the minimum acceptable SNR value to the acceptable percent error of the estimation of the latencies of the closure of heart valves with respect to ventricular depolarization. Figure 6 also shows that the dependence of the estimation error on the SNR is different for different heart valves. Those involved in the generation of the second tone (aortic and pulmonary valves) are more robust to a lower SNR than those responsible for the first tone (mitral and tricuspid valves).

We validated the presented results against a publicly available dataset. The validation shows that the minimum SNR values we computed on the sample population grant the corresponding uncertainty over the estimate in more than 90% of the recordings. We believe that this result allows us to demonstrate that our findings are independent of the sample population, the user, the recording system, and the context.

We believe that these original findings may have a relevant impact in designing devices to be used in a telemedicine context at the patient domicile, since they would allow the cardiologist to adapt the device to reject only those signals that are not of sufficient quality with respect to the specific condition of every single patient, thus maximizing the amount of reliable information collected. We consider these results as very promising and we believe they will facilitate the design of user-independent, low-energy, and reliable devices to be used in a telemedicine context to daily assess the condition of patients with a significant likelihood of developing acute heart failure episodes.

A possible limitation of the applicability of this method could be hypothesized considering that the threshold values of SNR herein reported have been obtained by applying the algorithm for the estimation of the latencies of heart sound components we previously published [4]. In fact, this algorithm includes a denoising step, discussed above in Section 2.2, which increases the SNR value of the original recording by a minimum of 3 dB up to a maximum of 14 dB. In fact, this is not a real limitation, since most processing techniques applied to PCG signals comprise denoising as a preprocessing step. Indeed, the denoising algorithm we adopted gives results that are similar to those reported by other Authors for state-of-the-art denoising algorithms applied to phonocardiography. In fact, other researchers reported an increase in SNR between 3 dB and 15 dB by using a variety of different algorithms [39,40,41,42,43]. Hence, since previous results reported in the literature for different PCG denoising algorithms and those we obtained by applying the algorithm described in Section 2.2 are very similar, we can hypothesize that applying a different noise reduction approach will not negatively impact the estimation of the minimum values of SNR that may be suitable to clinical applications.

One could argue that another obvious limitation of the proposed work is the unavailability of signals recorded on pathological subjects. Indeed, this choice was formed on purpose, to first test the approach on a normal population before extending it to pathological subjects. Moreover, this approach is imposed by most ethical committees. Nonetheless, we believe that the methodology herein presented is robust with respect to the health status of the subject, since it relies on simple numerical methods and on a very basic characteristic of the signal, instead of considering numerous signal features, which are more likely to change in the presence of a pathology.

The computational cost of the vast majority of the algorithms proposed in the literature makes them unsuitable for implementation in real time, even on 32-bit microprocessors with a floating-point processing unit. The approach herein presented may be easily implemented on high-end microprocessors and hence all the necessary data processing could be carried out within the detection device, without relying on an external computer or smartphone for computations.

To our best knowledge, only one previous research work implemented some quality assessment in a real-life monitoring application. In particular, Nemcova et al. [44] forecast a quality check on their app for blood pressure monitoring. The quality evaluation was performed visually by the user, by confirming that she/he could see periodical peaks within the signal shown on the screen.

We believe that numerous naïve users could be unable of providing a reliable opinion about the signal quality, even in an extremely simple way. This is particularly credible in our application, considering that the target users belong to the elderly population, since these subjects are most likely affected by CVDs.

Finally, it should be highlighted that more than half of the cited works related to PCG quality assessment [11,12,14,19,31,32,33,34,35,36,37,38,44] were published in the last 3 years, implying that this topic is gaining importance within the scientific community.

## 5. Conclusions

The aim of this work was to analyze the sensitivity to noise of the measurement of the latency of the components of the first and second heart sounds, estimated by means of an algorithm we previously developed [4]. We progressively added artificial noise to real PCG recordings to obtain, signals with a lower SNR. At each iteration, we estimated the four latency values associated to the closing of the mitral, tricuspid, aortic, and pulmonary valves.

We found that, in the worst case, recordings with an SNR as low as 14 dB are sufficient for obtaining a measurement uncertainty lower than 1 ms.

Since in most clinical applications clinicians can accept a measurement uncertainty substantially higher than 1 ms, we assessed the variation of the minimum acceptable SNR value as a function of the acceptable uncertainty. We concluded that the latencies of the atrioventricular valves are typically more sensitive to noise than their semilunar counterparts. An SNR equal to 10 dB is sufficient to obtain a 10% uncertainty on all the latencies.

Since the prolongation of the latency of the closing of heart valves is strictly related to a compromised ventricular function, which in turn may cause heart failure and other dysfunctions, evaluating valve timing through PCG is a promising technique, especially at the patient domicile in a telemedicine context. The user-independent evaluation of the quality of PCG signals is crucial for allowing the development of reliable systems for the home monitoring of patients prone to heart failure. Hopefully, this home monitoring approach should strongly reduce the need for patient hospitalization and possible negative outcomes.

## Figures and Tables

**Table 1:** Minimum acceptable SNR values (1-ms uncertainty). For each subject of the sample population, this table presents the initial SNR value and minimum acceptable SNR threshold for each latency. The overall SNR threshold is the maximum value obtained for each parameter. A “N/A” value (not available) is associated with signals where no threshold could be computed for a parameter because the condition was never met.

Subj ID	Initial SNR Values (dB)		Minimum Acceptable SNR Values (dB)			
	S_1_	S_2_	R-S_1,M_	R-S_1,T_	R-S_2,A_	R-S_2,P_
subj01	26	23	N/A	10	12	11
subj02	8	7	6	6	5	5
subj03	11	10	8	8	8	7
subj04	14	14	6	9	7	9
subj05	24	20	10	9	N/A	11
subj06	14	10	8	10	7	6
subj07	18	15	7	12	8	10
subj08	14	10	7	8	6	8
subj09	15	12	8	7	7	8
subj10	21	20	6	13	6	10
subj11	14	17	9	9	9	13
subj12	13	14	N/A	8	9	9
subj13	12	8	N/A	9	5	N/A
subj14	12	9	7	6	6	6
subj15	13	13	8	8	6	9
subj16	16	18	10	10	11	9
subj17	10	7	6	7	5	5
subj18	9	7	7	7	5	6
subj19	19	14	N/A	11	9	9
subj20	7	7	6	6	N/A	6
subj21	15	11	8	7	N/A	8
subj22	10	10	8	8	7	8
subj23	8	9	6	6	8	7
subj24	23	26	7	14	10	7
subj25	18	15	12	N/A	N/A	9
Minimum acceptable SNR values over the population			12	14	12	13

**Table 2:** Median, 25th percentile, and 75th percentile values for the four latencies over the sample population. The median value is used as reference for the computation of the percentage uncertainties.

	R-S_1,M_ (ms)	R-S_1,T_ (ms)	R-S_2,A_ (ms)	R-S_2,P_ (ms)
Median	44	77	368	392
25th percentile	38	69	353	377
75th percentile	60	96	393	432

## Data Availability

Data supporting the reported results were anonymized and privately stored on the data storage of the research group. Data are available upon request to the corresponding author.

## Supplementary Materials

The following are available online at https://www.mdpi.com/article/10.3390/s21217246/s1, Signals S1: matrix containing on the first row the recording performed on subj01 and on rows 2 to 6, five signals obtained from the above-mentioned recording through the described methodology, respectively with SNR equal to 25 dB, 20 dB, 15 dB, 10 dB, 5 dB. All signals are sampled at 1 kHz.
